# Multiple endocrine neoplasia type 1 with a frameshift mutation in its gene accompanied by a giant cervical lipoma and multiple fatty deposits in the pancreas: case report

**DOI:** 10.1186/s12902-021-00821-7

**Published:** 2021-08-12

**Authors:** Yoshiro Fushimi, Shinji Kamei, Fuminori Tatsumi, Junpei Sanada, Masashi Shimoda, Tomohiko Kimura, Atsushi Obata, Shuhei Nakanishi, Kohei Kaku, Tomoatsu Mune, Hideaki Kaneto

**Affiliations:** 1 Department of Diabetes, Endocrinology and Metabolism, Kawasaki Medical School, 577 Matsushima, 701-0192 Kurashiki, Japan; 2 Department of Diabetic Medicine, Kurashiki Central Hospital, Kurashiki, Japan

**Keywords:** MEN1, A giant cervical lipoma, Multiple fatty deposits in the pancreas

## Abstract

**Background:**

Multiple endocrine

neoplasia type 1 (MEN1) is a syndrome characterized by pituitary neoplasia, primary hyperparathyroidism and pancreatic endocrine tumor. Here we show a case of MEN1 with a germline frameshift mutation in its gene accompanied by a giant cervical lipoma and multiple fatty deposits in the pancreas.

**Case presentation:**

A 28-year-old man noticed the decreased visual acuity of both eyes and visited our institution. Since he was diagnosed as visual disturbance and brain computer tomography (CT) showed a mass in the pituitary fossa, he was hospitalized in our institution. Endoscopic trans-sphenoidal hypophysectomy and total parathyroidectomy with auto-transplantation were performed, and a giant cervical lipoma was resected. Furthermore, in genetic search, we found a germline frameshift mutation in MEN1 gene leading to the appearance of a new stop codon.

**Conclusions:**

We should bear in m

ind that giant skin lipoma and multiple abnormal fatty deposits in the pancreas could be complicated with MEN1.

## Background

Multiple endocrine neoplasia type 1 (MEN1) is a syndrome which is characterized by pituitary neoplasia, primary hyperparathyroidism and pancreatic endocrine tumor [1, 2]. MEN1 is one of the genetic diseases with autosomal dominant inheritance and its penetrance is high. It is known that various mutations in MEN1 gene are involved in the phenotype of MEN1 [3–5]. Among them, frameshift and nonsense mutations deform MEN1 protein structure and are thought to be pathological loss-of-function mutations. Here we show a case of MEN1 with a germline frameshift mutation in MEN1 gene who had a giant cervical lipoma and multiple abnormal fatty deposits in the pancreas.

## Case Presentation

**Fig. 1:**
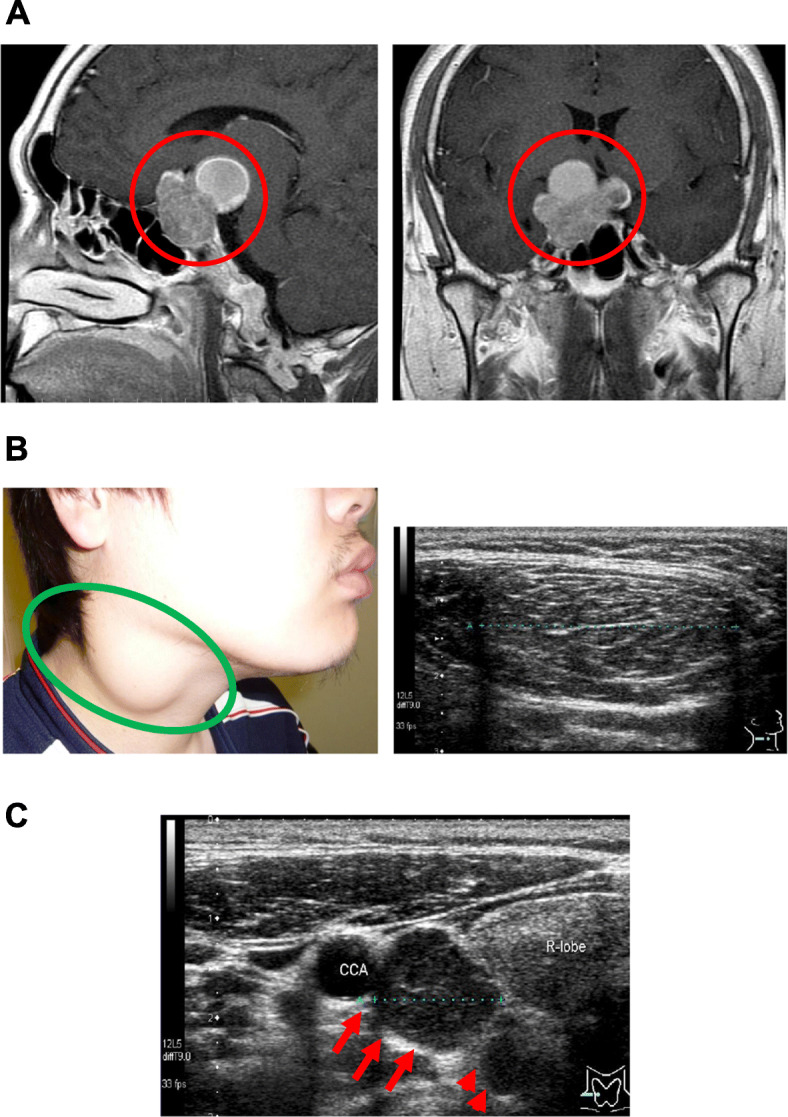
(**A**) Contrast-enhanced brain MRI. Neoplastic lesion was observed in the pituitary fossa (red circle). Infiltration of the tumor to all directions was observed. Its maximal diameter was approximately 50 mm, and it was a mixture of solid and cystic lesion. The cystic lesion seemed to be hematoma. Invasion of the cavernous sinus and compression of the optic nerve were also observed. (**B**) (Left panel) A large elastic mass was observed in right cervical region (green circle). (Right panel) The mass was clearly observed in neck ultrasonography. The view in this ultrasound image was transverse. (**C**) In neck ultrasonography, two parathyroid tumors were observed which maximal diameters were 38 mm (arrows) and 14 mm (arrow heads), respectively. The view in this ultrasound image was transverse

**Fig. 2:**
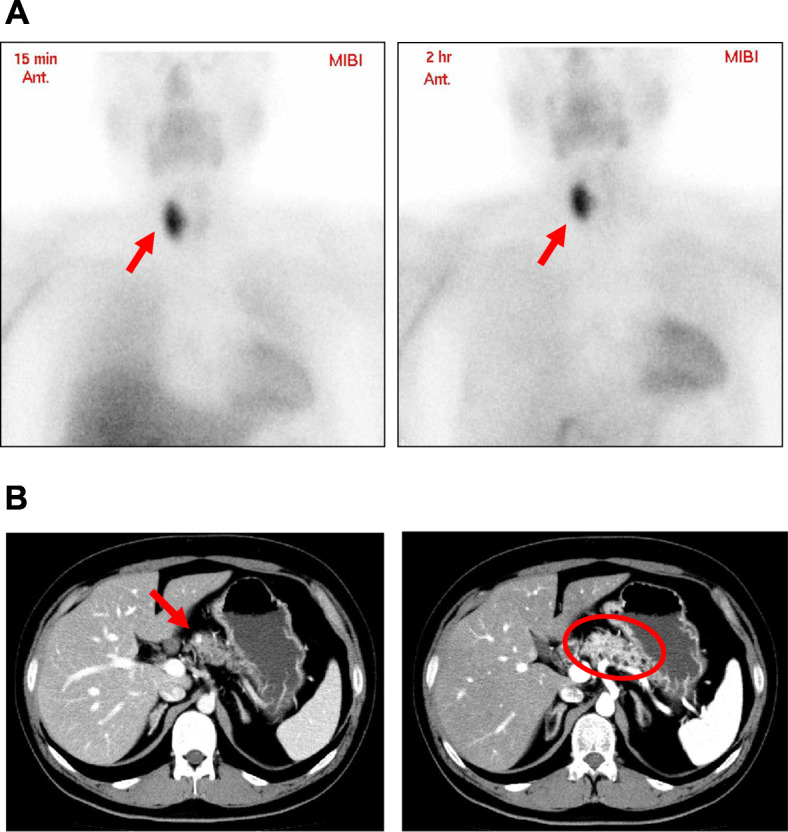
(**A**) In ^99m^Tc MIBI scintigraphy, MIBC accumulation was observed at 2 h as well as at 15 min in neck lesion which was thought to be parathyroid lesion (arrows in both panels). (**B**) Contrast-enhanced abdominal CT. Small tumor (diameter: 5 mm) was observed in the pancreatic head (an arrow in left panel). Multiple fat density areas were observed in the whole pancreas (a circle in right panel)

A 28-year-old man noticed the decreased visual acuity of both eyes and visited our institution. Since he was diagnosed as visual disturbance and brain computer tomography (CT) showed a mass in the pituitary fossa, he was hospitalized in our institution. On admission, his height and body weight were 171.4 cm and 83.7 kg. Blood pressure and heart rate were 113/63 mmHg and 63 bpm. Vision: (right) 30 cm manual valve, (left) 0.06. Field-of-view range: (right) nasal hemianopia, (left) normal. In contrast-enhanced brain magnetic resonance imaging (MRI), suspected neoplastic lesion was observed in the pituitary fossa (Fig. 1 A). Its maximal diameter was approximately 50 mm. It was a mixture of solid and cystic lesion which seemed to be hematoma. Invasion of the cavernous sinus and compression of the optic nerve were confirmed. In addition, large elastic mass was palpable in right cervical region, and the mass was isoechoic in neck ultrasonography (Fig. 1B). Intact-parathyroid hormone (PTH) level was high (273 pg/mL) and hypercalcemia (Ca, 12.1 mg/dL) were observed. Hyperprolactinemia (prolactin, 3,794.0 ng/mL) (normal range: 4.29–13.69 ng/mL) was also observed. Other endocrine hormone levels were within normal range as follows: ACTH, 59.9 pg/mL (7–63 pg/mL); cortisol, 13.5 µg/dL (7.07–19.6 mg/dL); DHEA-S, 388 µg/dL (85–690 mg/dL); GH, 0.08 ng/mL (0-2.47 ng/mL); IGF-1, 177 ng/mL (114–315 ng/mL); TSH, 1.90 µIU/mL (0.5-5.0 mIU/mL); FT3, 3.12 pg/mL (2.3–4.3 pg/mL); FT4, 0.52 ng/dL (0.9–1.7 ng/dL); LH, 0.8 mIU/mL (0.79–5.72 mIU/mL); FSH, 2.0 mIU/mL (2.0-8.3 mIU/mL); testosterone, 0.34 ng/mL (1.31–8.71 ng/mL). Hepatic dysfunction and dyslipidemia were observed: AST, 53 U/L; ALT, 102 U/L, γ-GTP, 69 U/L; LDL cholesterol, 145 mg/dL; HDL cholesterol, 44 mg/dL; triglycerides, 355 mg/dL. Neck ultrasonography showed two parathyroid tumors which maximal diameters were 38 mm and 14 mm, respectively (Fig. 1 C). In ^99m^Tc methoxyisobutylisonitrile (MIBI) scintigraphy, MIBC accumulation was observed in the neck lesion at 15 min and at 2 h which was thought to be the accumulation in the parathyroid lesion (Fig. 2 A). Furthermore, contrast-enhanced abdominal computer tomography (CT) showed a small tumor in the pancreas head (diameter: 5 mm) and multiple fat density lesions in the whole pancreas (Fig. 2B). These lesions were thought to be multiple abnormal fatty deposits in the pancreas. Various pancreatic hormone levels were almost within normal range as follows: glucagon, 137 pg/mL (reference range: 70–174 pg/mL); gastrin, 213 pg/mL (30–200 pg/mL); insulin, 18.4 µU/mL (2.2–12.4 µU/mL); trypsin, 340 ng/mL (100–550 ng/mL).

Based on these findings, we diagnosed this subject with MEN1 accompanied by pituitary neoplasia, primary hyperparathyroidism, a giant cervical lipoma and multiple abnormal fatty deposits in the pancreas. Cervical lipoma is considered as a cutaneous manifestation of the disease. Endoscopic trans-sphenoidal hypophysectomy was performed, and the treatment with carbergoline (1 mg/week) was performed for size reduction of residual pituitary tumor and the control of hyperprolactinemia. Such trans-sphenoidal intervention on the pituitary gland promptly resolved his visual disturbance. After then, prolactin level was gradually decreased to approximately 500 ng/mL, and there was no progression of pancreatic lesions in follow-up CT. After then, total parathyroidectomy with auto-transplantation and resection of cervical lipoma were performed. Furthermore, in genetic search, we found a germline frameshift mutation (c.1613delA in MEN1 gene (adenine deletion in exon 10)) in MEN1 gene in this subject which leads to the appearance of new stop codon at codon 588. This mutation was previously reported in another subject [6]. His parents were not genotyped at the MEN1 locus, but they did not have any symptom related to MEN1. Therefore, although MEN1 is known to be one of the genetic diseases, it is likely that this case is a propositus case. It is noted here that we cannot totally exclude the possibility this subject did not have family history because it is well known that there are many subjects who have MEN1 but do not have any symptoms even after the onset of the disease. About 1 year later, follow-up CT was taken, but there was no progression in pancreatic lesions.

Taken together, this subject with MEN1 had various characteristics including a germline frameshift mutation in MEN1 gene, the development of a giant cervical lipoma and multiple abnormal fatty deposits in the pancreas, and the absence of family history in addition to typical characteristics of MEN1 such as pituitary neoplasia and primary hyperparathyroidism.

## Discussion and conclusions

In this report, we showed a case of MEN 1 with a germline frameshift mutation (c.1613delA in MEN1 gene (adenine deletion in exon 10)) who was accompanied by a giant cervical lipoma and multiple abnormal fatty deposits in the pancreas. We assume it is likely that this frameshift mutation was causative in such alteration in the pancreas. Although it is known that cervical lipoma is sometimes observed in subjects with MEN1 and is considered as a cutaneous manifestation of the disease, we think that the cervical lipoma in this subject is very large compared to the reported ones. In addition, it seemed that the abnormal fat density lesions in the pancreas were multiple abnormal fatty deposits, although we cannot exclude the possibility that this lesion have or develop to another type of disease in the future. The tumors in the pancreas seemed to be non-functional at this point, but we think it is possible that these tumors would become malignant in the future and thus it would be necessary to continue to examine this point. Somatostatin receptor scintigraphy would be also useful to perform the localization and make a definitive diagnosis of possible neuroendocrine tumors in the pancreas. In addition, although we understood that this subject showed marked hypersecretion of PTH and hypercalcemia, the interval between drug therapy for prolactinoma and parathyroid operation was about 10 months. We explained the importance to this subject to take parathyroid operation, but he hesitated to have it. This was the reason why parathyroid operation was delayed.

Gastrin level in this subject (213 pg/mL) was slightly higher compared to its reference range (30–200 pg/mL). Since gastrin levels in subjects with gastrinoma are usually much higher, we think the possibility was quite low that this subject had gastrinoma. However, since we did not perform any secretion tests, we cannot completely exclude the possibility of gastrinoma. Insulin level in this subject (18.4 µM/mL) was also slightly higher compared to its reference range (2.2–12.4 µM/mL). We think that such hyperinsulinemia was induced by obesity. Since insulin levels in subjects with insulinoma are usually much higher and hypoglycemia was not observed at all in this subject, we think the possibility of insulinoma was quite low.

Lipoma is occasionally observed in subjects with MEN1; its frequency ranges from 5 to 34 % [7]. Therefore, we think that a giant cervical lipoma and multiple fatty deposits in the pancreas in this subject were associated with MEN1, although we cannot deny the possibility that there was no association between such alterations and MEN1. It has been thought that the pathogenesis of MEN1 is mainly brought out by the loss of heterozigosity (LOH) of MEN1 gene which is mapped on chromosome 11q13. Such mutation inactivates MEN1 tumor suppressor gene, which is involved in the regulation of DNA replication and repair. In addition, it has been thought that MEN1 gene mutation is also involved in the pathogenesis of less specific MEN1 tumors such as lipomas [7]. Germline inactivating mutations in the MEN1 gene that encodes menin predispose subjects to develop fatty change and/or tumors in various endocrine organs such as the pancreas and parathyroid [8]. Thereby, inactivation of menin in various MEN1-associated endocrine tissues facilitates epigenetic changes which finally leads to fatty change and tumorigenesis. It was also shown that the development of lipomas was associated with cytogenetic aberrations in the 12q13–q15 regions, although it is still controversial whether all are associated with them [7]. We assume that lipomas in subjects with MEN1 including that in the present subject was associated with cytogenetic aberrations in such regions. In general, lipomas are the most frequent benign soft tissue tumors and usually become symptomatic in case of compression of the surrounding structures. In this subject, a giant cervical lipoma was observed. To the best of our knowledge, such a giant lipoma and multiple fatty deposits in the pancreas are very rare in clinical practice.

While it was known that MEN1 gene product menin interacted with another important molecule checkpoint kinase 1 (Ches1), it was reported that some mutation in exon 10 of MEN1 gene disturbed the interaction between Menin and Ches1 and thereby augmented the possibility of endocrine tumor in the pancreas [9]. In this paper, the authors examined an association between MEN1 mutations in different interacting domains of Menin and the phenotype of pNENs in a retrospective analysis of a prospectively collected cohort of 71 genetically confirmed MEN1 patients. As the results, subjects with mutations leading to loss of interaction (LOI) with Ches1 interacting domain codons had significantly higher rates of functioning, malignant and aggressive pancreatic neuroendocrine neoplasias (pNENs) compared with subjects with mutations resulting in LOI with other domains. Furthermore, they showed that subjects with Ches1-LOI also had higher pNEN-related mortality. This case also had a frameshift mutation in exon 10 of MEN1 gene. Therefore, although speculative, we assume that the abnormality in the pancreas was associated with a frameshift mutation in exon 10 in this subject. From this point, we think that it is very important to perform very careful examination about the pancreas for a long time in this subject.

There are several limitations in this study. First, an ultrasound endoscopy with eventual biopsy could have clarified whether the abnormal fat density lesions in the pancreas were lipomas or small cysts, but we did not perform such biopsy due to high risk of biopsy from the pancreas. Second, 68 gallium scintigraphy could have helped us to understand the pathogenesis of this subject, but it was not performed in this case.

In conclusion, we should be bear in mind the possibility that a giant skin lipoma as well as multiple abnormal fatty deposits in the pancreas are accompanied by MEN1. In addition, we should know the possibility of MEN1 even when the subject does not have family history of MEN1 and does not have any symptom throughout the whole body. We think that the information in this case report would be useful and call for attention from the clinical point of view.

## Data Availability

Not applicable.

## Abbreviations

MEN1: Multiple endocrine neoplasia type 1
CT: Computer tomography
PTH: Parathyroid hormone
ACTH: Adrenocorticotropic hormone
DHEA-S: Dehydroepiandrosterone sulfate
GH: Groeth hormone
IGF-1: Insulin-like growth factor-1
TSH: Thyroid-stimulating hormone
FT3: Free triiodothyronine
FT4: Free thyroxine
ACTH: Adrenocorticotropic hormone
DHEA-S: Dehydroepiandrosterone sulfate
AST: Aspartate aminotransferase
ALT: Alanine aminotransferase
γ-GTP: γ-glutamyl transpeptidase
LDL: Low density lipoprotein
HDL: High density lipoprotein
MIBI: Methoxyisobutylisonitrile
